# Optimizing transitions of care antimicrobial prescribing at a community teaching hospital

**DOI:** 10.1017/ash.2023.504

**Published:** 2023-12-06

**Authors:** Jordan Smith, Jeremy Frens, Dhaval Mehta, Kushal Naik, Emily Sinclair, Tyler Baumeister

**Affiliations:** 1 Moses H. Cone Memorial Hospital, Greensboro, NC, USA; 2 Fred Wilson School of Pharmacy, High Point University, High Point, NC, USA

## Abstract

**Objective:**

Antibiotic prescribing at hospital discharge is an important focus for antimicrobial stewardship efforts. This study set out to determine the impact of a pharmacist-led intervention at hospital discharge on appropriate antimicrobial prescribing.

**Design:**

This was a pre-/post-study evaluating the impact of a pharmacist-led review on antibiotic prescribing at hospital discharge. Pharmacists evaluated antibiotic prescriptions at discharge for appropriate duration, spectrum of activity, frequency, and strength of dose. Each of these criteria needed to be met for an antibiotic regimen to be considered appropriate.

**Setting:**

Moses Cone Hospital is a 535-bed community teaching hospital in Greensboro, North Carolina.

**Patients or Participants:**

Patients ≥18 years of age discharged from the hospital with an antibiotic prescription were included. Exclusion criteria included patients discharged against medical advice, discharged to a skilled nursing facility, or prescribed indefinite prophylactic antimicrobial therapy.

**Interventions:**

A review of patients discharged with antibiotics in 2020 was performed. Patients discharged with antibiotic prescriptions from January 2021 to April 2022 were evaluated prior to discharge by pharmacists. The pharmacist made recommendations to providers based on their evaluations.

**Results:**

162 retrospective patients were screened, and 136 patients were screened at discharge from the hospital in the prospective cohort. 76/162 (47%) retrospective patients received appropriate antibiotic therapy at discharge, while 92/136 (68%) of prospective patients received appropriate discharge therapy (*p* = 0.001).

**Conclusions:**

In this study examining the efficacy of stewardship pharmacist intervention at hospital discharge, pharmacist review and recommendations were associated with an increased rate of appropriate antimicrobial prescribing.

**Ethics statement:**

This study was conducted under the approval of the Institutional Review Board of the Moses H. Cone Health System. The approval protocol number was 1483117-1 and took effect on September 2nd, 2019. As the research was either retrospective in nature or part of the standard of care recommendations, the project was granted expedited review.

## Introduction

Antimicrobial stewardship has been a cornerstone of hospital therapy for decades, and most institutions in the United States have some form of stewardship practice.^1^ Numerous publications have demonstrated the positive effects of antimicrobial stewardship on patient outcomes, antibiotic use, cost of therapy, and reduction in antibiotic-associated infections.^2^ Antibiotic use is prevalent in hospitals, with published data demonstrating that 40%–50% of patients receive antibiotics during their admissions.^3^ While antimicrobial stewardship efforts dedicated to patients during their hospital stays have received needed, beneficial attention, antibiotic prescriptions at hospital discharge are often left unaddressed by stewardship programs. To date, there are few publications addressing antibiotic stewardship at hospital discharge.

Patients treated for infections in the hospital are frequently discharged with time remaining in their antibiotic courses. Often, patients receive as many or more days of antibiotic therapy after discharge as they received during their hospital admission.^4^ Discharge prescriptions are frequently errant in dosage, duration, frequency, spectrum, or some combination of all of these factors.^5,6^ Studies have demonstrated that more than half of discharge antibiotic prescriptions contain at least some level of inappropriate therapy.^6^ Although inpatient stewardship efforts are prevalent, their successful implementation is not necessarily indicative of appropriate discharge antibiotic prescribing. Data suggest that prescribing of inappropriate fluoroquinolone antibiotics at discharge actually increased at institutions with successful inpatient fluoroquinolone restrictions.^7^ Given the data, there is good reason to focus efforts on antimicrobial stewardship at discharge.

Patient discharge is handled by a variety of parties, and the process can often be rushed for patients, prescribers, nurses, and discharge planners. Because of the difficulties inherent to the practice, antimicrobial stewardship at hospital discharge is much less frequently practiced and described in the literature than in-hospital stewardship. The process of discharging patients, or “transitions of care,” is an important element of the patient experience. Direct pharmacist involvement at transitions of care has demonstrated reductions in adverse event-related hospital visits, hospital readmissions, and emergency department visits.^8^ At Moses Cone Hospital, a community teaching hospital in Greensboro, North Carolina, we implemented a pharmacist-led transitions of care antimicrobial stewardship program in an effort to provide better discharge antibiotic prescribing for our patients. The purpose of this study was to evaluate the effectiveness of a stewardship-trained, clinical pharmacist-led discharge oral antibiotic program on the appropriateness of discharge antibiotic prescribing.

## Methods

### Research setting and timeline

Moses Cone Hospital is the flagship hospital of Cone Health in Greensboro, North Carolina. It is a 535-bed community teaching hospital. Pre-intervention data were collected over the calendar year 2020. Post-intervention data were collected from January 2021 to April 2022.

### Research design

This was a quasi-experimental, pre-/post-study evaluating appropriateness of antimicrobial prescribing at hospital discharge. The discharge antimicrobial stewardship intervention was implemented in January 2021. The intervention involved a pharmacist-led, multidisciplinary approach of antibiotic prescription auditing at hospital discharge. Pharmacists involved in this intervention were stewardship-trained with a specific focus on optimizing transitions of care. Pharmacists were alerted when patients were discharged with oral antibiotic prescriptions. They then reviewed patient charts for relevant disease information and provided feedback on appropriate discharge antibiotics for the discharge team. Specifically, pharmacists performed a daily review of hospitalized patients on antibiotic therapy. At discharge, if patients were to be discharged on antibiotics, a pended discharge antibiotic order was written by the pharmacist for the drug, duration, frequency, and end date of the prescription based on institutional or national guideline recommendations.^9-15^ Institutional guidelines for antibiotic choice and duration were available for community-acquired and hospital-acquired pneumonia, urinary tract infection, skin and soft tissue infection, intra-abdominal infection, and *Clostridioides difficile* colitis. Recommendations for therapy for bloodstream infections, osteomyelitis, and other infections were based on national guidelines as appropriate. When the orders for discharge were assembled, the prescriber could see the pharmacist’s recommendation and either sign the order or make changes based on their assessment. Data were collected to determine the appropriateness of antibiotic prescription. Appropriate prescribing included guideline-concordant indication, duration, spectrum, dose, and frequency of antibiotic administration. Infectious disease physicians were available for questions or discrepancies that arose and were available for consultation with discharge prescribers if they were needed.

### Data collection

Data collection for all patients included age, gender, comorbidities (diabetes mellitus, antibiotic use within the previous 6 months, chronic obstructive pulmonary disease, hospitalization within the previous 90 days, HIV infection, pregnancy, cirrhosis, history of multidrug-resistant organism, and end-stage renal disease), reason for admission, primary infectious disease diagnosis, hospital length of stay, infectious disease consultation, inpatient antibiotics administered, days of inpatient antibiotics received, indication for antibiotic treatment, discharge antibiotic regimen selected, days of discharge antibiotics prescribed, appropriateness of discharge antibiotic prescription, and percent of pharmacist recommendations accepted.

### Outcomes

The primary outcome of interest was appropriateness of antibiotic prescribing at discharge. Appropriate antibiotic therapy was defined as guideline-concordant indication, duration, spectrum, dose, and frequency of antibiotic administration.^16^ Appropriateness was determined independently by two stewardship pharmacists. Pharmacists evaluating appropriateness were not part of the discharge recommendation. If the pharmacists disagreed, the therapy was classified as inappropriate. Duration of therapy included number of days of inpatient antibiotics combined with the prescribed duration of outpatient antibiotics, and the appropriate duration was evaluated based on the disease being treated. Spectrum of activity appropriateness was determined by susceptibility of the offending pathogen(s) to the antibiotic(s) prescribed as well as the presence of any redundancies in therapy. If no susceptibility data were available, appropriateness was based on institutional guidelines for empiric therapy. Dose and frequency appropriateness were based on factors such as disease, patient variables, and guideline-driven dosing regimens.

Secondary outcomes included each individual component of appropriateness, i.e., duration, spectrum, dose, and frequency of antibiotics, days of excess antibiotic therapy, and number of pharmacist interventions accepted.

### Inclusion and exclusion criteria

Patients over the age of 18 discharged from the hospital with an oral antibiotic prescription were eligible for inclusion. Patients were excluded if they were discharged to a skilled nursing facility or hospice or if antibiotics were to be continued for chronic suppression or prophylaxis. These exclusion criteria were applied because the transitions of care pharmacist intervention was targeted to patients being discharged home on oral antibiotics for treatment of an acute infectious disease, and these patients did not fit eligibility criteria. Patients were identified for retrospective data collection with reports of patients discharged from the hospital on oral antibiotic prescriptions for the treatment of acute infectious diseases. Post-intervention patients were screened proactively for oral antibiotic therapy at discharge for the treatment of an acute infectious disease.

### Statistical analysis

Based on an estimated baseline rate of 50% appropriate discharge antimicrobial prescribing and assuming a type-1 error rate of 5% and 80% power, 194 patients in each arm were required to detect a 14% increase in appropriate antimicrobial prescribing. These assumptions on effectiveness were based on a previous study reported by Yogo, et al.^6^ 250 patients per cohort were targeted to ensure appropriate statistical power would be achieved. The primary outcome, a dichotomous outcome defined as overall appropriateness of discharge antimicrobial prescribing, was compared between pre- and post-intervention groups using a X^2^ test to evaluate whether the intervention was associated with increased appropriate antimicrobial antibiotics at discharge. Baseline and demographic variables, as well as secondary outcomes, were compared between the two groups using Student’s t-test, X^2^, or Fisher’s exact tests as appropriate. Multivariable logistic regression was performed with appropriate oral discharge antibiotic prescribing as the dependent variable. First, univariable regression was performed to determine the effects of the intervention as well as treatment and disease characteristics on the primary outcome of appropriate oral discharge therapy. Disease and treatment characteristics that were to be included in the multivariable model were preselected based on previous data suggesting they may have an influence on appropriate discharge antimicrobial prescribing.^17,18^ These characteristics included presence of pneumonia, urinary tract infection, skin and soft tissue infection, bloodstream infection, and/or intrabdominal infection. The presence of infectious disease consult and patient age were also included.^17,18^ Multivariable regression was then performed with appropriate oral discharge antibiotic prescribing as the dependent variable and each of the above-described elements as variables within the model. In the multivariable model, the outcome of interest was appropriate antimicrobial therapy, and odds ratios are reported for the impact of 1) the intervention, 2) pneumonia, 3) urinary tract infection, 4) skin and soft tissue infection, 5) bloodstream infection, 6) intra-abdominal infection, 7) infectious disease consult, and 8) patient age. For univariable and multivariable regression, odds ratios (OR) with confidence intervals (CI) were reported for likelihood of appropriate antibiotic prescribing at discharge based on the presence of the predictive variable. For example, an OR of 1.5 for appropriate antibiotic prescribing in the post-intervention group would indicate that the odds of receiving appropriate oral antibiotic at discharge are 1.5 times higher in the post-intervention compared to the pre-intervention group, and this same relationship holds true for all dichotomous variables collected and analyzed in the model. For continuous variables, eg, age, the OR reports the odds of the outcome occurring given each 1-unit increase (year of age in this case) in the variable. *P*-values <0.05 were considered statistically significant. SPSS version 29 was used for all analyses.

## Results

162 patients were included in the pre-intervention group, and 136 patients were included in the post-intervention group. Demographic information as well as pathogen and treatment characteristics are provided in Table 1. Demographic characteristics were similar between the groups.

**Table 1.** Patient, pathogen, and treatment characteristics

Patient, pathogen, and treatment characteristics (*N* = 298)						
		Pre (*N* = 162)		Post (*N* = 136)		
Characteristics		*N*	%	*N*	%	*p*
Age	Average (SD)	62.3	17.1	65.9	16.5	0.067
Women		72	44.4%	75	55.1%	0.066
Race	White	101	62.3%	89	65.4%	0.58
	Black/African American	54	33.3%	40	29.4%	0.468
	Asian	3	1.9%	3	2.2%	1
	Hispanic/Latino	2	1.2%	2	1.5%	1
	Prefer not to disclose	2	1.2%	2	1.2%	1
Comorbidities	Diabetes	65	40.1%	48	35.3%	0.392
	COPD	32	19.8%	34	25.0%	0.277
	Cirrhosis	3	1.9%	4	2.9%	0.706
	CKD	49	30.2%	31	22.8%	0.148
	Chronic liver disease	2	1.2%	2	1.5%	1
	CHF	44	27.2%	32	23.5%	0.474
	HIV	2	1.2%	2	1.5%	1
	Other immunosuppression	22	13.6%	19	14.0%	0.922
	Pregnancy	2	1.2%	1	0.7%	1
	No comorbidities	27	16.7%	20	14.7%	0.644
MDRO risks	Hosp. previous 90 days	27	16.7%	51	37.5%	0.001
	Abx. previous 180 days	37	22.8%	38	27.9%	0.312
	MDRO history	8	4.9%	8	5.9%	0.719
ID consult		5	3.1%	22	16.2%	0.001
Culture Obtained?		104	64.2%	115	84.6%	0.001
	Blood	83	51.2%	97	71.3%	0.001
	Tissue	20	12.3%	13	9.6%	0.445
	Sputum/BAL	10	6.2%	9	6.6%	0.876
	Other	48	29.6%	63	46.3%	0.003
Organism		104	64.2%	115	84.6%	0.001
	MSSA	9	5.6%	0	0.0%	0.001
	MRSA	5	3.1%	6	5.2%	0.89
	*S. pneumoniae*	0	0.0%	1	0.9%	1
	*E. faecalis*	3	1.9%	2	1.7%	0.67
	*E. coli*	8	4.9%	21	18.3%	0.021
	*K. pneumoniae*	3	1.9%	8	7.0%	0.221
	*Proteus spp.*	0	0.0%	2	1.7%	0.499
	*P. aeruginosa*	0	0.0%	3	2.6%	0.248
	Other	23	14.2%	32	27.8%	0.33
	No organism growth	53	51.0%	40	34.8%	0.016
Inpatient antibiotics	Cefazolin	21	13.0%	16	11.8%	0.755
	Cephalexin	12	7.4%	6	4.4%	0.28
	Ampicillin	0	0.0%	3	2.2%	0.094
	Ampicillin-sulbactam	9	5.6%	20	14.7%	0.008
	Amoxicillin	3	1.9%	7	5.1%	0.195
	Amox-Clav	12	7.4%	20	14.7%	0.043
	Pip-Tazo	22	13.6%	20	14.7%	0.781
	Ceftriaxone	61	37.7%	77	56.6%	0.001
	Cefdinir	12	7.4%	10	7.4%	0.986
	Cefepime	25	15.4%	40	29.4%	0.004
	Meropenem	1	0.6%	3	2.2%	0.334
	Ciprofloxacin	5	3.1%	12	8.8%	0.033
	Levofloxacin	7	4.3%	6	4.4%	1
	Azithromycin	33	20.4%	35	25.7%	0.272
	Clindamycin	10	6.2%	2	1.5%	0.072
	Doxycycline	25	15.4%	18	13.2%	0.591
	Linezolid	1	0.6%	7	5.1%	0.026
	Vancomycin	57	35.2%	62	45.6%	0.068
	Daptomycin	0	0.0%	2	1.5%	0.207
	TMP-SMX	4	2.5%	4	2.9%	1
	Metronidazole	22	13.6%	40	29%	0.001
	Oral vancomycin	0	0.0%	7	5.1%	0.004
	Other	15	9.3%	16	11.8%	0.480
Antibiotic indication	Bloodstream infection	6	3.7%	21	15.4%	0.001
	Pneumonia	66	40.7%	39	28.7%	0.03
	UTI	14	8.6%	21	15.4%	0.069
	SSTI	53	32.7%	15	11.0%	0.001
	Osteomyelitis	3	1.9%	3	2.2%	1
	IAI	12	7.4%	4	2.9%	0.088
	CDI colitis	0	0.0%	21	15.4%	0.001
	Other	8	4.9%	12	8.8%	0.182
Discharge antibiotics	Cephalexin	26	16.0%	17	12.5%	0.385
	Cefdinir	20	12.3%	21	15.4%	0.44
	Amoxicillin	4	2.5%	9	6.6%	0.081
	Amox-Clav	31	19.1%	28	20.6%	0.754
	Azithromycin	18	11.1%	13	9.6%	0.662
	Clindamycin	9	5.6%	1	0.7%	0.024
	Doxycycline	29	17.9%	10	7.4%	0.007
	TMP-SMX	10	6.2%	5	3.7%	0.326
	Linezolid	2	1.2%	7	5.1%	0.085
	Ciprofloxacin	7	4.3%	8	5.9%	0.539
	Levofloxacin	9	5.6%	4	3.7%	0.445
	Metronidazole	5	3.1%	9	6.6%	0.151
	Oral vancomycin	0	0.0%	18	13.2%	0.001
	Other	12	7.4%	3	2.2%	0.060

Note. SD, standard deviation; COPD, chronic obstructive pulmonary disease; CKD, chronic kidney disease; CHF, congestive heart failure; HIV, human immunodeficiency virus; MDRO, multidrug-resistant organism; Hosp, hospital; Abx, antibiotics; ID, infectious diseases; BAL, bronchoalveolar lavage; MSSA, methicillin-susceptible *Staphylococcus aureus*; MRSA, methicillin-resistant *Staphylococcus aureus*; *S. pneumoniae*, *Streptococcus pneumoniae*; *E. faecalis*, *Enterococcus faecalis*; *E. coli*, *Escherichia coli*; *K. pneumoniae*, *Klebsiella pneumoniae*; spp., species; *P. aeruginosa*, *Pseudomonas aeruginosa*; TMP-SMX, trimethoprim-sulfamethoxazole; UTI, urinary tract infection; SSTI, skin and soft tissue infection; IAI, intra-abdominal infection. *P*-value <0.05 considered statistically significant.

Regarding pathogen and treatment characteristics, there were some important differences between our cohorts. Compared to the pre-intervention group, infectious disease consultation was more prevalent post-intervention (3.1% vs 16%, respectively, *p* = 0.001), more patients in the post-intervention group were hospitalized within the preceding 90 days (16.7% vs 37.5%, respectively, *p* = 0.001), and cultures were more likely to be obtained in the post-intervention group (64.2% vs 84.6%, respectively, *p* = 0.001). Several antibiotics were used with different frequency between the two groups, and these data are fully reported in Table 1. Significantly more patients in the retrospective group were treated for pneumonia (40.7% vs 28.7%, respectively, *p* = 0.03) and skin and soft tissue infection (32.7% vs 11.0%, respectively, *p* = 0.001). Fewer patients in the retrospective group were treated for bloodstream infection (3.7% vs 15.4%, respectively, *p* = 0.001) and *Clostridioides difficile* colitis (0.0% vs 15.4%, respectively). In the retrospective group, more patients were treated for diseases that have institutional guidelines in place compared to the post-intervention group (90.1% vs 73.5%, *p* < 0.001). Full data, including descriptions of infectious disease diagnoses, antibiotic indications, and antibiotics prescribed at discharge, are listed in Table 1.

Patients in the pre-intervention group were associated with a lower likelihood of optimal oral antibiotic therapy at discharge compared to the post-intervention group. (46.9% vs 67.6%, respectively, *p* = 0.001, Table 2). Reasons for inappropriate therapy were similar between the pre-intervention and post-intervention groups, with inappropriate duration accounting for the majority of inappropriate prescribing in both groups (86.0% and 95.5%, respectively). When comparing days of excess prescribed antibiotic therapy between the groups among patients who were given inappropriate durations, the pre-intervention group received an average of 4.6 excess days compared to an average of 2.6 excess days in the post-intervention group (*p* = 0.001).

**Table 2.** Outcomes

Outcomes (*N* = 298)						
		Pre (*N* = 162)		Post (*N* = 136)		
Outcome		*N*	%	*N*	%	*p*
Appropriate therapy		76	46.9%	92	67.6%	0.001
Inappropriate therapy		86	53.1%	44	32.4%	0.001
	Inappropriate duration	74	86.0%	42	95.5%	0.102
	Inappropriate spectrum	12	14.0%	3	6.8%	0.228
	Inappropriate dose	7	8.1%	2	4.5%	0.445
	Inappropriate frequency	11	12.8%	1	2.3%	0.05
Average inappropriate days	(Total, Mean)	334	4.6 avg	110	2.6 avg	0.001
Interventions accepted				90	66.1%	–

Note. *P*-value <0.05 considered statistically significant.

Regression analyses were performed to determine the association of the intervention with appropriate discharge antimicrobial therapy. Other variables included in regression analyses included patient age, infectious disease consultation, the presence of bloodstream, urinary tract, skin and soft tissue, intra-abdominal, or respiratory infection. These variables were selected based on previously published data suggesting their association with appropriate discharge antimicrobial therapy.^17,18^ In univariable analyses, intervention (OR 2.37, *p* < 0.001), infectious disease consult (OR 2.95, *p* = 0.028), pneumonia (OR 2.35, *p* < 0.001), and skin and soft tissue infection (OR 0.30, *p* < 0.001) were associated with a difference in likelihood of optimal discharge antibiotic therapy. In the multivariable model, pharmacist intervention (OR 2.15, *p* = 0.007), infectious disease consult (OR 2.81, *p* = 0.044), and skin and soft tissue infection (OR 0.41, *p* = 0.041) were associated with a difference in likelihood of optimal discharge antibiotic therapy. Full data are available in Table 3.

**Table 3.** Univariable and multivariable regression

Effect of specified variables on the likelihood of optimal discharge antimicrobial therapy						
	Optimal discharge antibiotic therapy		Univariable		Multivariable	
	*N*	%	OR (95% CI)	*p-value*	OR (95% CI)	*p-value*
Overall	168/298	56.4%	–	–	–	–
Post-intervention	92/136	67.6%	2.37 (1.47-3.80)	<0.001	2.15 (1.24-3.75)	0.007
Age (SD)	64.0 (16.6)	N/A	0.99 (0.99-1.01)	0.865	1.01 (0.99-1.02)	0.328
ID consult	21/27	77.8%	2.95 (1.15-7.55)	0.028	2.81 (1.03-7.70)	0.044
Abx indication						
BSI	14/27	51.9%	0.82 (0.37-1.81)	0.620	0.57 (0.21-1.58)	0.280
Pneumonia	73/105	69.5%	2.35 (1.42-3.89)	<0.001	2.02 (0.90-4.49	0.087
UTI	20/35	57.1%	1.04 (0.51-2.11)	0.922	0.95 (0.37-2.44)	0.952
SSTI	23/68	33.8%	0.30 (0.17-0.53)	<0.001	0.41 (0.17-0.96)	0.041
IAI	7/16	43.8%	0.59 (0.21-1.61)	0.30	0.73 (0.22-2.46)	0.612

Note. Odds ratios reflect the odds of optimal discharge antibiotic therapy given the presence of the variable of interest, eg, post-intervention, age, ID consult, BSI, pneumonia, UTI, SSTI, or IAI, compared to the absence of those variables. Univariable regression accounts for the variable of interest only, while multivariable regression accounts for all of these variables and reports their contributions to the odds ratio. OR, odds ratio; CI, confidence interval; SD, standard deviation; ID, infectious diseases; BSI, bloodstream infection; UTI, urinary tract infection; SSTI, skin and soft tissue infection; IAI, intrabdominal infection; Abx, antibiotics. *P*-value <0.05 considered statistically significant.

## Discussion

In this quasi-experimental study evaluating patients discharged from the hospital on antibiotics, involvement of a pharmacist at hospital discharge was associated with an increased likelihood of appropriate antibiotic prescribing. Even after accounting for factors previously demonstrated to impact appropriate antibiotic prescribing at discharge, pharmacist intervention maintained a strong association with appropriate antibiotic use as patients were sent home with antibiotic prescriptions.

It is well established that unnecessary antibiotic prescribing facilitates increased risk of adverse effects, multidrug-resistant organisms, and *Clostridioides difficile* colitis.^19^ Often, patients are prescribed courses of antibiotic therapy that exceed those recommended in clinical guidelines, and numerous recent studies have also demonstrated that perhaps even shorter courses of antibiotic therapy can often be used.^20^ Although institutions—including ours—frequently perform well on inpatient antimicrobial stewardship metrics, discharge therapy is frequently rife with inappropriate antibiotic selection, dosing, and especially duration.^4-6,18^ This was indeed the case in this study, as over half of the patients in the pre-intervention group were prescribed discharge antibiotic therapy inappropriately. With a dedicated, daily review of patients being discharged on antibiotics, pharmacist intervention was able to increase the rate of appropriate antibiotic prescribing at discharge to nearly 68% of patients, a significant difference. Multivariable regression in this study included covariates previously demonstrated to be associated with prolonged antibiotic therapy at discharge consisting of pneumonia, intra-abdominal infection, urinary tract infection, skin and soft tissue infection, bloodstream infection, patient age, and infectious disease consult.^17,18^ Even after accounting for these variables, pharmacist intervention was associated with a 2.15 increased odds of appropriate antimicrobial prescribing compared to patients who received no intervention.

Similar to previous studies, the data here demonstrated that unnecessarily prolonged durations of therapy were the largest driver of inappropriate prescribing. However, among patients who were treated inappropriately in this study, the amount of unnecessary days of antibiotic therapy was reduced from an average of 4.6 days to 2.6 days.

Previous studies have suggested that pharmacist intervention can have a positive impact on discharge antimicrobial prescribing. Foolad and colleagues demonstrated a significant improvement in appropriate duration of therapy for patients treated for community-acquired pneumonia, and they also saw no difference in clinical outcomes such as mortality, readmission, or *Clostridioides difficile* colitis.^21^ Similarly, Mercuro and colleagues were able to demonstrate increased appropriateness of antibiotic prescribing at discharge in patients with pneumonia, urinary tract infection, skin and soft tissue infection, and intra-abdominal infection.^17^ This study demonstrated similar success, and the program was not resource-intensive beyond a commitment from the pharmacist team and administration. If institutions are committed to providing discharge oversight for antibiotic prescriptions, the demands upon their inpatient stewardship teams would be minimal. Although this study took place at only one hospital within Cone Health, the program has since expanded into multiple health system sites based on the results.

There are limitations to this study. The patient groups were unequal in size, and 194 patients were not able to be included in each group. As such, the study did not achieve statistical power as defined in the methods. It was difficult to enroll enough patients who were discharged with oral antimicrobial therapy and who also underwent stewardship pharmacist review in the timeline established. In the post-intervention group, even with an additional ∼4 months of data collection, only 136 patients were enrolled. However, given the large difference in appropriate therapy between the groups of 21%, a statistically significant difference was able to be established. Although the study was underpowered, a positive effect of the intervention was still able to be established.

There was some heterogeneity among the patient groups. Differences in rates of diagnoses among the groups included pneumonia, bloodstream infection, skin and soft tissue infection, and *Clostridioides difficile* colitis. However, the multivariable model included all of these disease states except *Clostridioides difficile* colitis, and the intervention still demonstrated an increased odds of 2.15 optimal antibiotic prescribing at discharge. Also, there were actually statistically more pneumonia patients in the pre-intervention group, and pneumonia on its own was associated with an increased likelihood of appropriate antibiotic therapy. If anything, this would have biased results toward a higher likelihood of optimal prescribing in the pre-intervention group. And because all cases of *Clostridium difficile* colitis were treated in the prospective arm, data were analyzed without those cases to ensure those infections did not have a disproportionate impact on appropriate prescribing. Without *Clostridioides difficile* colitis included, appropriate antibiotic therapy remained 46.9% in the pre-intervention group, and appropriate antibiotic therapy actually increased to 67.0% in the post-intervention group (*p* < 0.001). There was also heterogeneity among antibiotics prescribed within the hospital and at hospital discharge, which is understandable given the various disease states evaluated in this study. In an effort to limit any differences that may have come from the different antibiotic choices, this study was designed to describe as appropriate any antimicrobial therapy that fit within institutional or national guidelines. Data on relevant clinical outcomes such as hospital readmission, adverse drug events, or mortality were not collected. This was outside the scope of the study as proposed, and this study was conducted with the assumption pharmacist involvement as described would not impact patient mortality. In a similar study, Mercuro and colleagues were able to demonstrate that patient outcomes were either unimpacted or positively impacted by pharmacist intervention, and shorter courses of antibiotics at discharge were associated with the intervention.^17^ Given these previously reported data and the positive association with antibiotic prescribing demonstrated, there is little concern that this intervention would have negatively impacted patients in any way.

## Conclusion

This transitions of care pharmacist intervention successfully demonstrated an association with improved discharge oral antibiotic prescribing. Similar efforts can be undertaken in other hospitals and health systems. These data suggest that pharmacist intervention at hospital discharge can have a positive impact on outpatient discharge oral antibiotic prescribing.

## Research transparency and reproducibility

De-identified data from this project are available for analysis at request.
